# MicroRNA-144 suppresses osteosarcoma growth and metastasis by targeting ROCK1 and ROCK2

**DOI:** 10.18632/oncotarget.3305

**Published:** 2015-04-13

**Authors:** Wei Wang, Xin Zhou, Min Wei

**Affiliations:** ^1^ Department of Orthopaedic Surgery, Renji Hospital, School of Medicine, Shanghai Jiaotong University, Shanghai 200127, China

**Keywords:** miR-144, ROCK1, ROCK2, osteosarcoma, metastasis

## Abstract

Osteosarcoma (OS) is the most common primary tumor of bone. MicroRNAs (miRNAs) are a class of endogenously expressed small non-coding RNAs that are strongly implicated in cancerous processes. However, our current understanding of the biological role of miRNAs in OS remains incomplete. In the present study, miR-144 was markedly downregulated in OS cell lines and clinical specimens. Low-level expression of miR-144 was significantly associated with distant metastasis and poor prognosis. Functional studies demonstrated that ectopic expression of miR-144 suppresses tumor cell proliferation and metastasis *in vitro* as well as *in vivo*. Furthermore, we identified *Rho-associated kinases 1* and *2* (*ROCK1* and *ROCK2*) as direct targets for miR-144 binding, resulting in suppression of their expression. Exogenous expression of ROCK1 or ROCK2 in 143B-miR-144 cells partially restored miR-144-inhibited cell proliferation and invasion. In clinical OS specimens, ROCK1 and ROCK2 levels were elevated, relative to that in paired normal bone tissues, and inversely correlated with miR-144 expression. Taken together, miR-144 suppresses OS progression by directly downregulating ROCK1 and ROCK2 expression, and may be a promising therapeutic target for OS.

## INTRODUCTION

Osteosarcoma (OS) is the most common primary malignant tumor arising from bone in children and young adults, with high biologic aggressiveness [1]. Despite significant advances in treatment options (generally comprising a combination of surgery and multiagent chemotherapy), the clinical outcomes and prognosis of OS patients remains poor [2, 3]. Metastasis remains one of the most enigmatic aspects of the disease. The majority of OS patients with lung metastases are not suitable for surgery, resulting in low 5-year survival rates of ≤ 30%. In contrast, the 5-year survival rate for patients with localized disease is >60% [4, 5]. Clarification of the molecular mechanisms governing carcinogenesis and the metastatic processes of osteosarcoma is therefore critical for the reduction of mortality.

Accumulating evidence has shown that cancer progression and metastasis involve microRNAs (miRNAs), short non-coding RNA molecules that inhibit multiple target mRNAs through binding to their 3′-untranslated regions (UTR) in a sequence-specific manner [6, 7]. miRNA alterations and dysfunction play critical roles during tumorigenesis and metastasis via regulation of cancer cell proliferation, differentiation, apoptosis, migration and invasion [8–12]. Indeed, aberrant miRNA expression in OS has been shown to contribute to cancer development and metastasis by promoting oncogene expression or inhibiting tumor suppressor genes [13, 14]. For example, miR-20a encoded by the miR-17–92 cluster increases the metastatic potential of osteosarcoma cells by regulating Fas expression [15]. Downregulation of miR-143 promotes osteosarcoma cell invasion through MMP-13 upregulation [16], and silencing of miR-133a reduces the malignancy of CD133^high^ osteosarcoma-initiating cell population through restoring the expression of multiple target genes (SGMS2, UBA2, SNX30, and ANXA2) [17]. However, although miRNAs have been extensively investigated in recent years, their roles in OS development and as potential markers for diagnosis and prognosis remain unclear.

In the current study, we examined the potential function of miR-144 in OS. We first analyzed the expression of miR-144 in human OS cells and tissues and tested its effect on cell proliferation, migration and invasion. Moreover, we investigated a potential role of miR-144 on OS tumorigenesis and metastasis in a murine model. Finally, we explored the molecular mechanism underlying the function of miR-144 in OS. Our study will provide a better understanding of OS development and progression.

## RESULTS

### miR-144 expression is downregulated in OS cell lines and tissues

**Figure 1:**
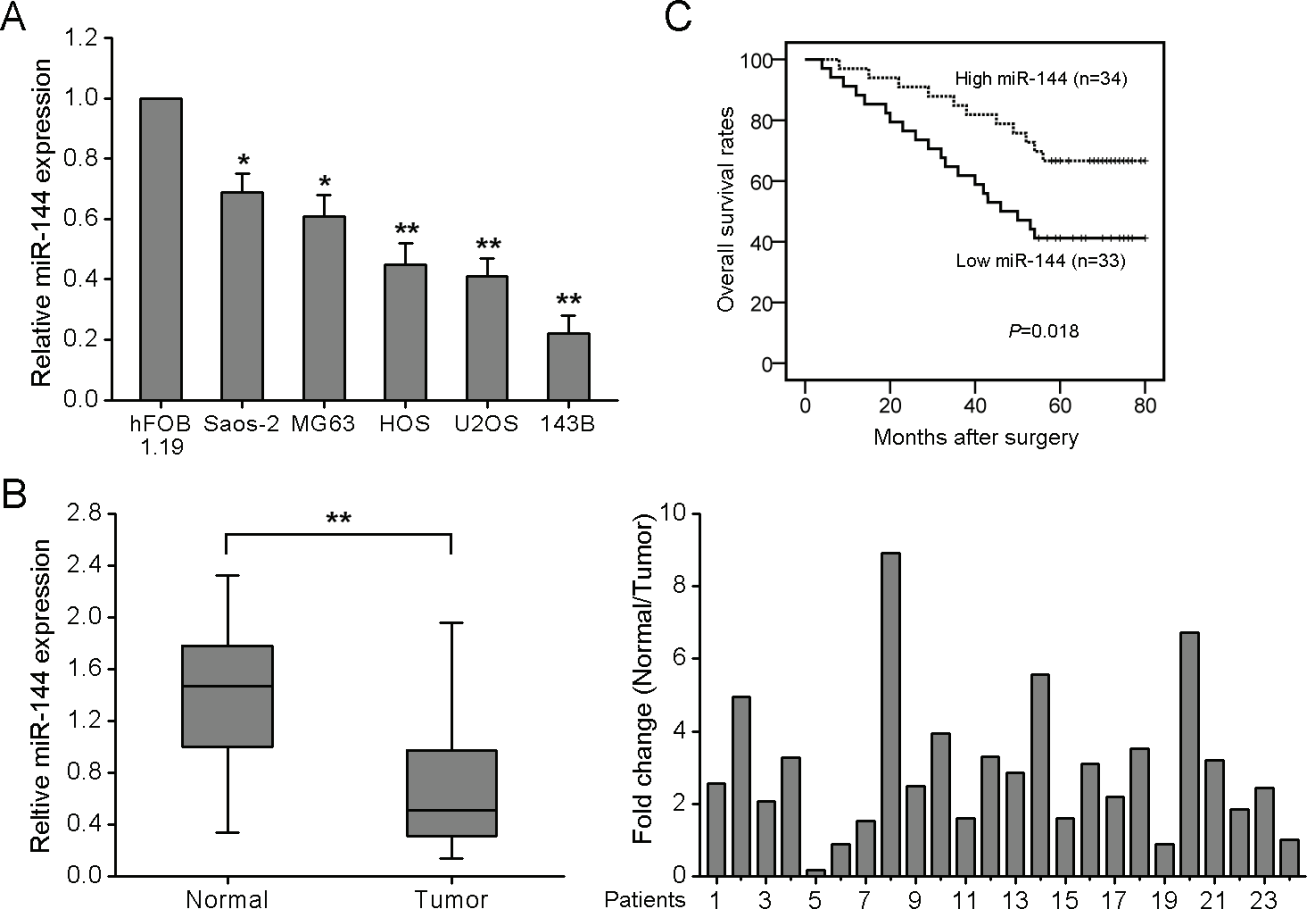
Downregulation of miR-144 in OS cell lines and tissues is associated with poor prognosis **A.** qRT-PCR was conducted to quantify endogenous expression of miR-144 in the human osteoblastic cell line, hFOB 1.19, and four OS cell lines, using U6 as the normalization control. **B.** miR-144 expression is frequently decreased in OS tissues. The left-hand panel shows expression of miR-144 in 24 paired OS and adjacent normal bone tissues. The right-hand panel shows miR-144 expression relative to adjacent normal tissues. Each bar represents the mean of 3 independent experiments. **C.** Kaplan-Meier analysis for overall survival in 67 OS patients in high- and low-risk groups based on miR-144 expression levels. *P < 0.05, **P < 0.01.

Initially, miR-144 expression was examined in four OS cell lines using quantitative real-time PCR (qRT-PCR). All OS cell lines tested displayed lower levels of miR-144 than the normal human osteoblastic cell line, hFOB 1.19 (Fig. 1A). Downregulation of miR-144 was also observed in clinical specimens, as evident from qRT-PCR analysis of 24 pairs of human primary OS tumors and adjacent normal bone tissues. In 66.7% (16 of 24) of the primary OS tissues, miR-144 expression was decreased by at least 2-fold (N/T ≥ 2-fold), compared to the adjacent non-tumor tissues (Fig. 1B).

### miR-144 expression is associated with poor prognosis of OS patients

To determine the clinicopathologic significance of miR-144 aberrations, levels of miR-144 were quantified in a cohort of 67 archived OS tissues using qRT-PCR. The median value of miR-144 expression was used as the cut-off point for dividing tumors into two groups: low expression (<median, *n* = 33) and high expression group (>median, *n* = 34). Low expression of miR-144 was significantly associated with distant metastasis, while no significant association was observed for other parameters (Supplementary Table 3). Kaplan-Meier analysis revealed longer survival times for patients with high miR-144 than those with low miR-144 expression (Fig. 1C). Our results suggest that downregulation of miR-144 contributes to OS pathogenesis, supporting its application as a prognostic biomarker predictive of better outcome for this disease.

### miR-144 suppresses OS cell proliferation *in vitro*

**Figure 2:**
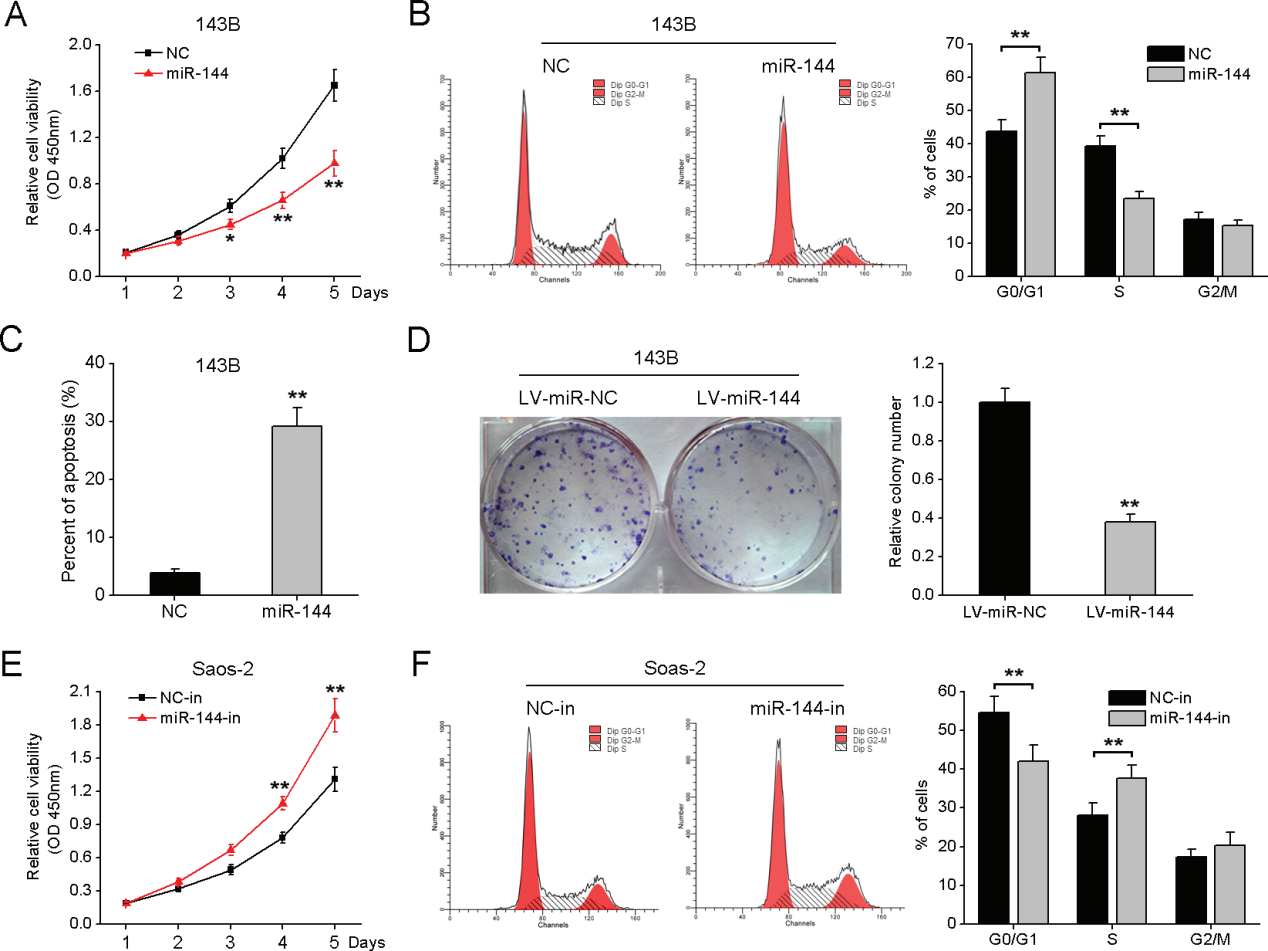
miR-144 suppresses OS cell proliferation *in vitro* **A.** 143B cells were transiently transfected with miR-144 mimics or NC mimics, and cell viability determined with the CCK-8 assay. **B.** FACS analysis of 143B cells was performed after transfection with miR-144 mimics or NC mimics. **C.** The apoptosis cells were stained with Annexin V and PI, and analyzed using FACS. **D.** Representative photographs and quantitative analysis of plate colony formation of 143B cells stably expressing miR-144. Silencing of miR-144 in Saos-2 cells significantly promoted proliferation **E.** and cell cycle G1/S transition **F.** *P < 0.05, **P < 0.01.

To explore the potential role of miR-144 in OS pathogenesis, 143B cells with high metastatic potential and low endogenous miR-144 expression, were transfected with miR-144 mimics, and miR-144 expression assayed using qRT-PCR (Supplementary Fig. 1A). Data from the cell viability assay showed that upregulation of miR-144 significantly suppresses the proliferation of 143B cells, compared with miR-NC-transfected cells (Fig. 2A). Flow cytometry analysis revealed that miR-144 overexpression leads to an increased percentage of cells in the G1 phase, alone with a decrease in S-phase cells (Fig. 2B), suggesting that this miRNA induces G1/S arrest. Moreover, the rate of apoptosis was significantly higher in 143B cells overexpressing miR-144 (Fig. 2C). The lentivirus system expressing miR-144-GFP was additionally applied to generate 143B-miR-144 cells stably expressing miR-144 (Supplementary Fig. 1B). As expected, the colony formation rate of these cells was significantly decreased (Fig. 2D).

In loss-of-function experiments using miR-144 inhibitors, silencing of miR-144 in Saos-2 cells with low metastatic potential and high endogenous miR-144 levels led to a significant increase in cell proliferation and increased percentage of cells in the S-phase (Supplementary Fig. 1C, Fig. 2E and 2F). These results clearly demonstrate that miR-144 regulates OS cell proliferation through effects on cell cycle distribution.

### miR-144 suppresses OS cell migration and invasion *in vitro*

**Figure 3:**
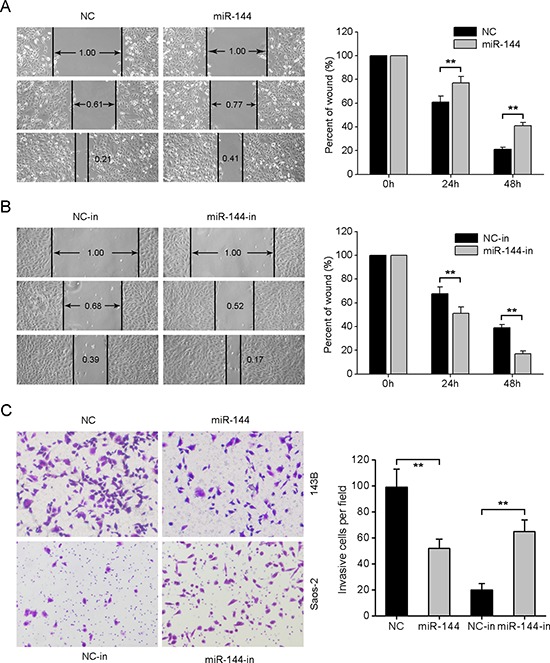
miR-144 suppresses OS cell migration and invasion *in vitro* **A.** Wound-healing assay for 143B cells transfected with miR-144 mimics or NC mimics. **B.** Wound healing assay for Saos-2 cells transfected with miR-144 inhibitors or NC inhibitors. **C.** Representative images (left) and quantification (right) of the Transwell invasion assay in the indicated cells. *P < 0.05, **P < 0.01.

We further investigated whether miR-144 weakens the migratory and invasive capabilities of OS. In the wound healing assay, miR-144 re-expression suppressed the migration of 143B cells to a significant extent (Fig. 3A). Conversely, the migration of Saos-2 cells was enhanced when endogenous miR-144 was silenced with specific inhibitors (Fig. 3B). Similarly, the Matrigel invasion assays showed that upregulation of miR-144 led to significantly decreased invasive ability of 143B cells, whereas silencing of miR-144 induced a marked increase in Saos-2 cell invasion (Fig. 3C). Our findings support the ability of miR-144 to attenuate the migration and invasion of OS cells.

### Upregulation of miR-144 suppresses tumor growth and metastasis in mice

**Figure 4:**
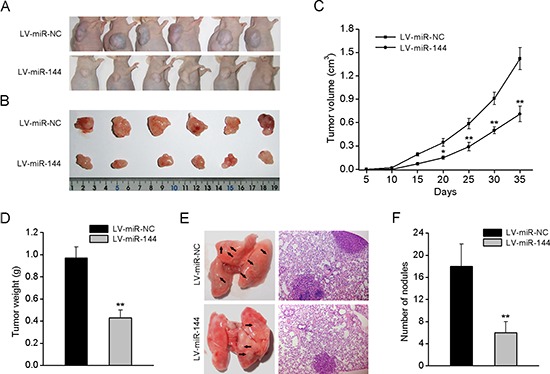
miR-144 suppresses tumorigenesis and metastasis in nude mice **A.** 143B cells stably overexpressing miR-144 or NC were inoculated subcutaneously into nude mice (*n* = 6). At 35 days post inoculation, mice were sacrificed. Representative tumors are shown in **B. C.** Growth curve of tumor volumes. **D.** Tumor weight. **E.** Representative photos and H&E-stained sections of lung tissues isolated from mice administered a tail vein injection of 143B cells stably overexpressing miR-144 or NC (*n* = 6). Quantification of lung metastatic nodules of each group is shown in **F.** *P < 0.05, **P < 0.01.

To explore the relationship between miR-144 and tumorigenesis *in vivo*, 143B cells stably overexpressing miR-144 were injected subcutaneously into nude mice and the animals were monitored closely for tumor growth. As shown in Fig. 4A, tumors formed in miR-144-overexpressing cells grew slower than those in cells expressing the control vector. In addition, miR-144-overexpressing tumors were smaller in size and had lower tumor volume and weight compared to the controls (Fig. 4B–4D).

To further determine the effects of miR-144 on tumor metastasis, nude mice were injected with 143B cells expressing miR-144 via the tail vein. At six weeks after injection, mice were anesthetized, their lungs were dissected, and H&E staining performed to evaluate tissue morphology (Fig. 4E). The number of lung metastasis nodules was significantly decreased in the 143B-miR-144 group, compared with control group (Fig. 4F), confirming that overexpression of miR-144 inhibits the metastasis of OS cells *in vivo*.

### miR-144 directly targets ROCK1 and ROCK2

**Figure 5:**
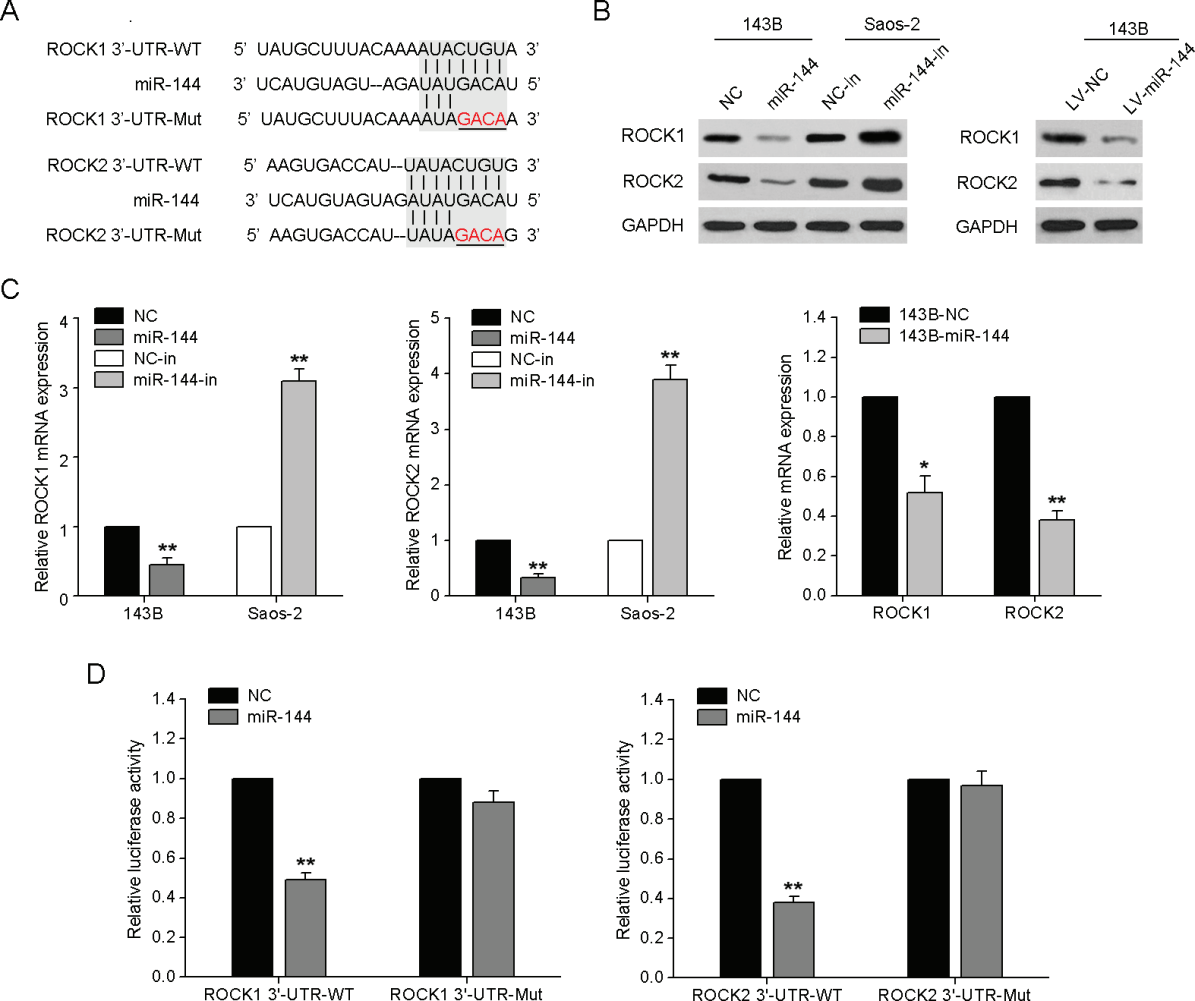
*ROCK1* and *ROCK2* are direct downstream targets of miR-144 **A.** Binding sites of miR-144 in the 3′-UTR regions of *ROCK1* and *ROCK2*. A mutant miR-144 binding site was generated in the complementary site for the seed region of miR-144. **B.** Detection of ROCK1 and ROCK2 proteins in 143B and Saos-2 cells after transfection with miR-144 mimics/inhibitors or miR-144 lentivirus infection. **C.** Detection of *ROCK1* and *ROCK2* mRNA in 143B and Saos-2 cells. **D.** miR-144 mimics or NC mimics, and a pGL3 luciferase vector containing the wild-type or mutant *ROCK1* or *ROCK2* 3′-UTR were co-transfected into 143B cells, and the relative luciferase activity was measured. *P < 0.05, **P < 0.01.

To elucidate the mechanisms underlying miR-144-mediated suppression of OS pathology, we searched for potential target genes of miR-144 using the bioinformatics algorithms miRanda and TargetScan. *ROCK1* and *ROCK2*, both containing a miR-144-binding site in the 3′-UTRs (Fig. 5A), were selected for further experimental validation, in view of their critical roles in tumor cell proliferation and invasion [18]. We further examined whether ROCK1 and ROCK2 levels are negatively regulated by miR-144 in OS cell lines. As shown in Fig. 5B and 5C, mRNA and protein levels of both ROCK1 and ROCK2 were significantly reduced or enhanced in response to miR-144 mimics and miR-144 inhibitors in 143B and Saos-2 cells, respectively, compared to the corresponding controls. Analogously, in 143B cells stably overexpressing miR-144, expression levels of these target genes were similarly decreased. We also confirmed elevated miR-144 with reduced ROCK1 and ROCK2 protein in miR-144-overexpressing tumors of nude mice (Supplementary Fig. 2).

To ascertain whether miR-144 interacts with the 3′-UTRs of *ROCK1* and *ROCK2*, the miR-144-based luciferase assay was performed. As expected, miR-144 directly bound to wild-type *ROCK1* and *ROCK2* 3′-UTRs, leading to significantly reduced luciferase activities, whereas cells with mutant *ROCK1* or *ROCK2* 3′-UTR displayed much higher luciferase activities (Fig. 5D). Based on these results, we conclude that *ROCK1* and *ROCK2* are direct downstream targets of miR-144 in OS cells.

### Downregulation of ROCK1 or ROCK2 is a key step in the tumor repressor function of miR-144

The above findings prompted us to investigate whether miR-144 suppresses OS growth and metastasis through inhibitory effects on ROCK1 and ROCK2. For this purpose, expression of ROCK1 and ROCK2 was initially restored in miR-144-overexpressing 143B cells via transfection of constructs containing *ROCK1* or *ROCK2* ORF without the 3′-UTR (Supplementary Fig. 3A). Functional studies revealed that ectopic expression of *ROCK1* or *ROCK2* partially, but significantly, promoted cell proliferation, G1/S transition, cell invasion impaired by miR-144 (Supplementary Fig. 3B–3D). On the other hand, silencing of *ROCK1* or *ROCK2* via transfection of specific siRNA in 143B cells significantly inhibits cell proliferation, induces G1 arrest, and inhibits cell invasion, mimicking the biological effects of miR-144 overexpression (Supplementary Fig. 3B–3D). Evidently, downregulation of ROCK1 or ROCK2 constitutes a critical step in the tumor suppressor activity of miR-144.

### ROCK1 and ROCK2 are upregulated in OS and inversely correlated with miR-144 expression

**Figure 6:**
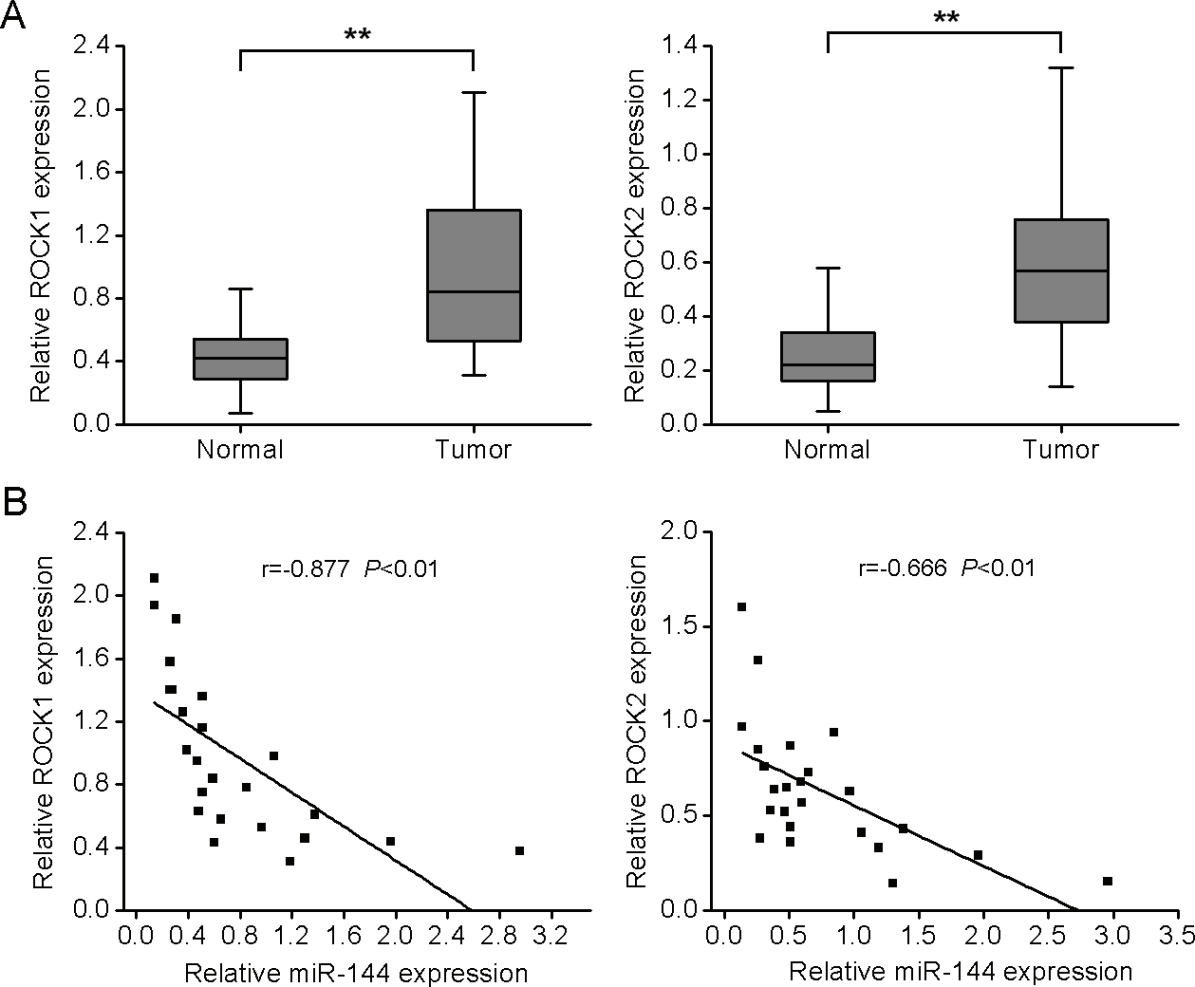
ROCK1 and ROCK2 are upregulated in OS specimens and inversely correlated with miR-144 levels **A.** ROCK1 and ROCK2 mRNA levels in 24 fresh OS specimens and adjacent normal bone tissues. **B.** Spearman's correlation analysis between miR-144 expression and ROCK1 or ROCK2 mRNA level. *P < 0.05, **P < 0.01.

Finally, ROCK1 and ROCK2 expression levels were measured in OS specimens and adjacent normal bone tissues. qRT-PCR analysis showed significantly higher mRNA levels of both *ROCK1* and *ROCK2* in OS, compared with normal bone tissue (Fig. 6A). Similarly, enrichment of ROCK1 and ROCK2 proteins were detected chiefly in tumor tissues, relative to normal bone tissues (Supplementary Fig. 4). Spearman's correlation analysis disclosed an inverse correlation between miR-144 expression and that of *ROCK1* and *ROCK2* (Fig. 6B).

## DISCUSSION

Dysregulation of miRNAs is reported in many human cancers [19]. Therefore, improved understanding of the gene networks orchestrated by miRNAs may provide more effective biomarkers and therapeutic targets for cancer patients. In the present study, we presented preliminary evidence that miR-144 plays a negative regulatory role in OS growth and metastasis via targeting *ROCK1* and *ROCK2*.

Differential expression of miR-144 has been detected in several human cancers. Specifically, miR-144 is downregulated in colorectal cancer [20], bladder cancer [21], non-small cell lung carcinoma [22] and papillary thyroid carcinoma [23], but upregulated in nasopharyngeal carcinoma [24] and esophageal cancer [25]. Consistent with data from previous microarray analyses [26], miR-144 was dramatically downregulated in OS tissues and cell lines, compared with their normal counterparts in the current study. Low expression of miR-144 was associated with distant metastasis and poor prognosis in OS patients, indicating that downregulation of miR-144 may be a critical step in OS progression.

There are two conflicting views on the function of miR-144 in human cancers. Iwaya *et al*. initially showed that downregulation of miR-144 is associated with colorectal cancer progression via activation of mTOR signaling pathway [20]. Consistently, Guo and co-workers reported that miR-144 suppression induces an increase in bladder cancer cell proliferation through targeting EZH2 and regulating Wnt signaling [21]. However, in nasopharyngeal carcinoma, miR-144 is reported to possess oncogenic properties based on its ability to promote cell proliferation [24]. Data from the current study showed that miR-144 overexpression leads to inhibition of *in vitro* cell growth and invasion, induction of cell cycle arrest and apoptosis, and suppression of *in vivo* tumor growth and metastasis. Conversely, downregulation of miR-144 promoted cell growth, invasion, and cell cycle transition to S phase, supporting the view that miR-144 mainly acts as a tumor suppressor in OS.

Generally, miRNAs function as posttranscriptional repressors that exert biological activities by suppressing their target genes [19]. To date, several targets of miR-144 have been identified, including Klfd [27], Rac1 [28], ZFX [22], mTOR [20], PTEN [24], Versican [29], ABCA1 [30], ADAM10 [31], EZH2 [21]. Using bioinformatics analysis, we further identified *ROCK1* and *ROCK2* as putative miR-144 targets in this study. Both mRNA and protein levels of these molecular targets were significantly downregulated in miR-144-expressing cells. In luciferase reporter assays, miR-144 overexpression led to a significant decrease in luciferase reporter activities of cells expressing ROCK1 and ROCK2 with wild-type but not mutant 3′-UTRs.

Rho-associated kinase (ROCK) is an essential downstream effector of the Rho small GTPase, which acts as a molecular switch that binds GTP (active) and GDP (inactive) to regulate cell survival, proliferation and cytoskeleton organization, inducing alterations in cell shape/morphology, adhesion and movement [32–34]. Two existing isoforms, ROCK1 and ROCK2, are known. Increased expression of ROCK is well documented in tumors, and related to cancer progression, metastasis and poor prognosis [35–38]. Conversely, inhibition of ROCK induces cell death, disrupts angiogenesis and reduces metastasis in several tumor types, including OS [18, 38, 39]. Therefore, ROCK proteins are currently under consideration as therapeutic targets for cancer. In a previous study, we demonstrated that ROCK1 is significantly upregulated in OS tissues, compared with normal bone tissues, and repressed by miR-340, resulting in suppression of OS cell proliferation and invasion *in vitro* and *in vivo* [40]. In addition, Zucchini *et al*. reported that CD99 suppresses osteosarcoma cell migration through inhibition of ROCK2 activity [41]. All these data indicate that ROCKs are promising targets for treatment of OS.

Several miRNAs have been shown to directly target ROCK mRNAs to exert their biological functions. For example, Zheng and colleagues showed that miR-148a downregulates ROCK1 to suppress tumor cell invasion and metastasis in gastric cancer [42]. Consistently, Shen *et al*. reported that miR-139 suppresses metastasis and progression of hepatocellular carcinoma by downregulating Rho-kinase 2 [43]. In OS, ROCK1 is also a target of several other miRNAs, including miR-340, 335, 145 [40, 44, 45]. However, the specific roles of ROCK2 and related miRNAs in OS remain to be established. Our results indicate that both ROCK1 and ROCK2 are functional targets of miR-144. Both isoforms were significantly overexpressed, and their mRNAs levels were inversely correlated with miR-144 expression in clinical specimens. Overexpression of *ROCK1* or *ROCK2* partially, but significantly, antagonized the inhibitory effect of miR-144. Moreover, downregulation of *ROCK1* or *ROCK2* significantly led to inhibition of cell proliferation and invasion, mimicking the biological effects of miR-144 overexpression.

In conclusion, decreased miR-144 expression in OS is closely associated with disease progression and metastasis. Furthermore, miR-144 appears to function as a tumor suppressor in the development and progression of OS via downregulation of ROCK1 and ROCK2. Accordingly, we propose that following further clarification of the specific role of miR-144, this miRNA may be a promising therapeutic target for OS.

## MATERIALS AND METHODS

### Human tissue samples

OS tissue samples (*n* = 67) were collected between 2008 and 2010 at Renji Hospital, Shanghai Jiaotong University School of Medicine, and 24 OS tissues and matched adjacent normal bone tissues obtained between 2011 and 2013. None of the patients had received chemotherapy prior to surgery. All tissue biopsies were immediately frozen in liquid nitrogen at the time of surgery and stored at −80°C until use. The clinical features of OS patients are shown in Supplementary Table 1. For the use of clinical materials for research purposes, prior patient consent was obtained, and the study was approved by the Ethics Committee of Renji Hospital, School of Medicine, Shanghai Jiaotong University.

### Cell culture

Normal human osteoblasts, hFOB 1.19, and four human osteosarcoma cell lines, Saos-2, MG-63, HOS, U2OS and 143B, were purchased from American Type Culture Collection (Manassas, VA, USA). Cell lines were cultured in Dulbecco's modified Eagle's medium (DMEM) (Gibco, Carlsbad, CA) supplemented with 10% heat-inactivated FBS, 100 U/ml penicillin and 100 mg/ml streptomycin at 37°C in a humidified incubator containing 5% CO_2_.

### Quantitative real-time PCR (qRT-PCR)

Total RNA was extracted from cell lines and tumor specimens using TRIzol reagent (Invitrogen, Carlsbad, CA). RNA quantity and integrity were assessed using Nanodrop and Agilent 2100 Bioanalyzer systems, respectively. cDNA was synthesized from total RNA using the PrimeScript RT reagent Kit (TaKaRa, Dalian, China) according to the manufacturer's instructions. Real-time PCR was performed using SYBR Premix Ex Taq II (TaKaRa) in the ABI PRISM 7900 Sequence Detection System (Applied Biosystems, Foster City, CA). The level of mature miR-144 was normalized relative to U6 endogenous control and *ROCK1* and *ROCK2* expression was normalized relative to *β-actin* (endogenous control) using the 2^−ΔΔCT^ method. The primers used were all purchased from RiBoBio (Guangzhou, China) and shown in Supplementary Table 2.

### Lentivirus infection and oligonucleotide transfection

Lentiviral vectors expressing miR-144 and scrambled RNA were purchased from GeneChem (Shanghai, China). 143B cells were infected with recombinant lentiviruses plus 8 μg/ml Polybrene (Sigma, St Louis, MO). miR-144 mimics (sense 5′-UACAGUAUAGAUGAUGUACU-3′, anti-sense 5′-UACAUCAUCUAUACUGUAUU-3′), miR-144 inhibitor (5′-AGUACAUCAUCUAUACUGUA-3′) or scrambled oligodeoxynucleotides were synthesized and purified by RiboBio. siRNA for ROCK1 and ROCK2 were obtained from Sigma. Transfection of miRNA was performed using Lipofectamine RNAiMAX (Invitrogen) according to the manufacturer's instructions. Transfection of ROCK1 and ROCK2 siRNAs was using Lipofectamine 2000 reagent (Invitrogen).

### Plasmid construction

The coding sequences of *ROCK1* and *ROCK2* were amplified and cloned into pcDNA3.1(+) to generate pcDNA3.1(+)-*ROCK1* and pcDNA3.1(+)-*ROCK2* plasmids. The primer sequences used were as follows: ROCK1-F: 5′-CACGATATCATGTCGACTGGGGACAGTTT-3′, ROCK1-R: 5′-CATGCGGCCGCTTAACTAGTTTTTCCAGATG-3′; ROCK2-F: 5′-CTAGCTAGCATGAGCCGGCCCCCGCCGACGG-3′, ROCK2-R: 5′-CGCGGATCCTTAGCTAGGTTTGTTTGGGGCA-3′. The correct PCR products were confirmed by DNA sequencing. The 3′UTRs of human *ROCK1* and *ROCK2* were amplified from OS cell line cDNAs using PCR, and cloned into a pGL3 vector (Promega, Shanghai, China). The primers used for amplification were: ROCK1-3′UTR-F: 5′-ATCGGTACCAGTCTGTGACTACAAAATAT-3′, ROCK1-3′UTR-R: 5′-GCCAAGCTTATCACAAATGTCTTTACCAG-3′; ROCK2-3′UTR-F: 5′-CATGGTACCCAGTGATATTGACTGCATCT-3′, ROCK2-3′UTR-R: 5′-CGCAAGCTTTATATGTGTGTGTGTTTATA-3′. For mutagenesis of the miR-144-binding site, the QuikChange site-directed Mutagenesis Kit (Agilent Technologies, Palo Alto, CA, USA) was employed according to the manufacturer's instructions. Transfection of plasmids was performed using Lipofectamine 2000 reagent.

### Luciferase reporter assay

Cancer cells were seeded in 24-well plates the day before transfection, followed by co-transfection with the constructed pGL3 vector (100 ng), 5 ng pRL-SV40 Renilla luciferase construct (for normalization), and 100 ng miR-144 mimics or control. Luciferase activity was measured after 48 h using the Dual luciferase reporter assay system (Promega). *Renilla* luciferase activity was normalized to that of firefly luciferase. Experiments were repeated at least three times.

### Cell proliferation, colony formation, cell cycle and apoptosis assays

Cell proliferation was evaluated using Cell Counting Kit-8 (Dojindo, Kumamoto, Japan). Cells (2 × 10^3^) were seeded in 96-well plates and cultured for 1, 2, 3, 4 and 5 days after transfection. CCK-8 solution (10 μl) was added to each well, followed by incubation for 2 h. Absorbance was measured at 450 nm with a Microplate Autoreader (Bio-Rad, Hercules, CA). Experiments were repeated at least three times. For the colony formation assay, cells were seeded in 6-well plates at a density of 500 cells per well and cultured for two weeks. Colonies were stained with 1% crystal violet for 30 s after fixation with 4% paraformaldehyde for 5 min, photographed and counted. Experiments were repeated at least three times. For cell cycle analysis, cells were seeded in 6-well plates at a density of 2 × 10^5^ cells per well. At 48 h after transfection, cells were detached via trypsinization, washed three times with ice-cold PBS and resuspended in 80% ethanol for at least 8 h at −20°C. Cells were fixed and stained with 50 μg/ml propidium iodide (Keygen, Nanjing, China). Cell cycle distribution was analyzed using FACSCaliber (BD Bioscience, MA, USA). For apoptosis detection, transfected cells were stained with AnnexinV/PI double staining kit (BD biosciences, Bedford, MA) according to the manufacturer's protocol. Apoptotic cells were examined by flow cytometry.

### Wound healing assay

Wound healing assays were performed to detect cell migration. Cells were seeded in 6-well plates and allowed to reach confluence. An artificial wound was made using a 200 μl pipette tip across the cell monolayer. Cells were rinsed with PBS and cultured in DMEM. Wound closure was observed at 0, 24 and 48 h, and imaging performed under a microscope.

### Cell invasion assay

Transwell invasion assays were performed according to the manufacturer's protocol (BD Biosciences). Briefly, transfected cells (1 × 10^5^) in serum-free medium were added to the top chamber coated with Matrigel and incubated at 37°C in a humidified incubator containing 5% CO_2_. Cells that invaded the lower chamber were stained with 10% crystal violet (Sigma) and quantitated by counting in five different areas under a light microscope.

### Western blotting

Total protein was extracted by lysing cells in RIPA buffer containing protease inhibitor. Lysates were incubated on ice for 10 min and centrifuged at 8, 000 × g to remove cellular debris. Protein samples were separated using 10% SDS-PAGE and transferred to a PVDF membrane. The membrane was blocked with 5% non-fat milk in TBS-T for 4 h at room temperature, and incubated with the primary antibodies against ROCK1 and ROCK2 (Abcam, Cambridge, UK). Blots were washed and incubated with HRP-conjugated secondary antibodies (Invitrogen). GAPDH (Abcam) was employed as an internal reference. Immunoreactive bands were detected using the enhanced chemiluminescence system according to the manufacturer's protocol (Pierce, Rockford, IL).

### Xenograft nude mouse model

Four-week-old BALB/c nude mice were provided by the Experimental Animal Center of Shanghai Jiaotong University School of Medicine (Shanghai, China). All animal experiments were approved by the Animal Care and Use Committee of Shanghai Jiaotong University School of Medicine. 143B-vector or 143B-miR-144 cells were injected subcutaneously into the flanks of mice (1 × 10^6^ cells per animal). Tumor size was measured every 5 days, and tumor volumes (mm^3^) calculated using the formula: V = L × W^2^/2, where L and W represent the longest and shortest diameters, respectively. All mice were killed 35 days after seeding of tumor cells, and the tumor weights measured. For the *in vivo* tumor metastasis assay, 2 × 10^6^ 143B-vector or 143B-miR-144 cells were injected into the lateral tail veins of nude mice. After six weeks, whole lungs were resected and tumor nodules counted. Lung tissues were fixed in 10% neutral phosphate-buffered formalin and embedded in paraffin. The metastatic nodules were counted by gross and microscopic examination.

### Statistical analysis

Each experiment was repeated at least three times. All data are expressed as means ± s.d. The Kaplan-Meier method was used to calculate the survival curve, and log-rank test to determine statistical significance. The differences between groups were analyzed using Student's *t*-test or χ2 analysis. Spearman's correlation tests were used to analyze the association between miR-144 and *ROCK1* or *ROCK2* mRNA expression in OS tissues. All statistical analyses were performed using SPSS 15.0 software (SPSS Inc., Chicago, IL). *P* < 0.05 was considered statistically significant.

## SUPPLEMENTARY FIGURES AND TABLES


