# Supplementary material for: MicroRNA-144 suppresses osteosarcoma growth and metastasis by targeting ROCK1 and ROCK2
Source: Oncotarget. 2015 Apr 13;6(12):10297–308. doi: 10.18632/oncotarget.3305 (PMC4496356; doi:10.18632/oncotarget.3305)
Supplement: Supplementary file 1 [file oncotarget-06-10297-s001.pdf]

SUPPLEMENTARY FIGURES AND TABLES

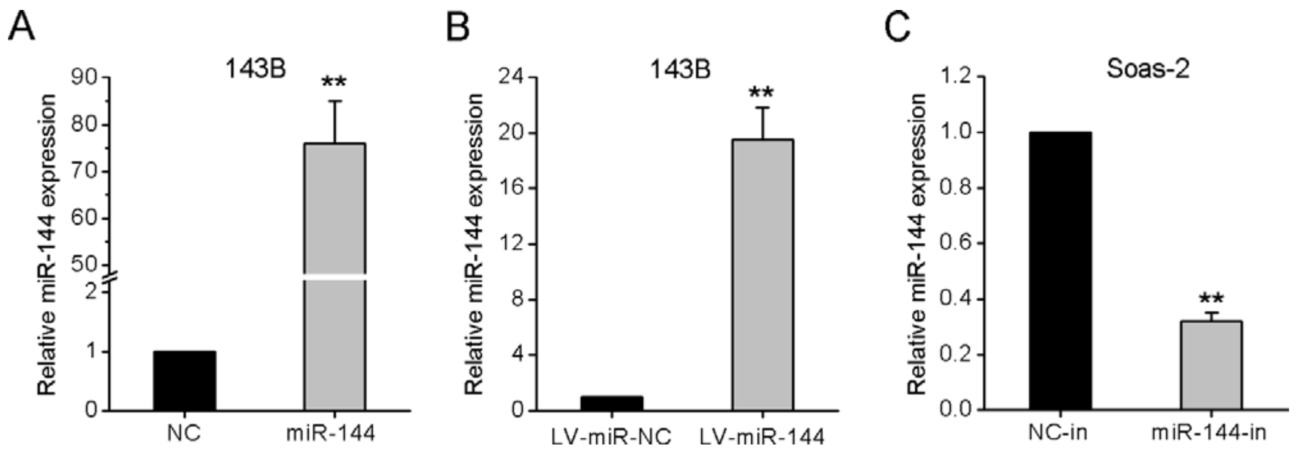

Supplementary Figure S1: Modification of miR-144 levels in the OS cell lines 143B and Soas-2. A. Relative expression of miR-144 in 143B cells transfected by miR-144 mimics (100 nM) or NC mimics. **B.** Relative expression of miR-144 in 143B cells stably infected with lentivirus expressing miR-144 or NC. **C.** Relative expression of miR-144 in Saos-2 cells transfected by miR-144 inhibitor or NC inhibitor. ***P* < 0.01.

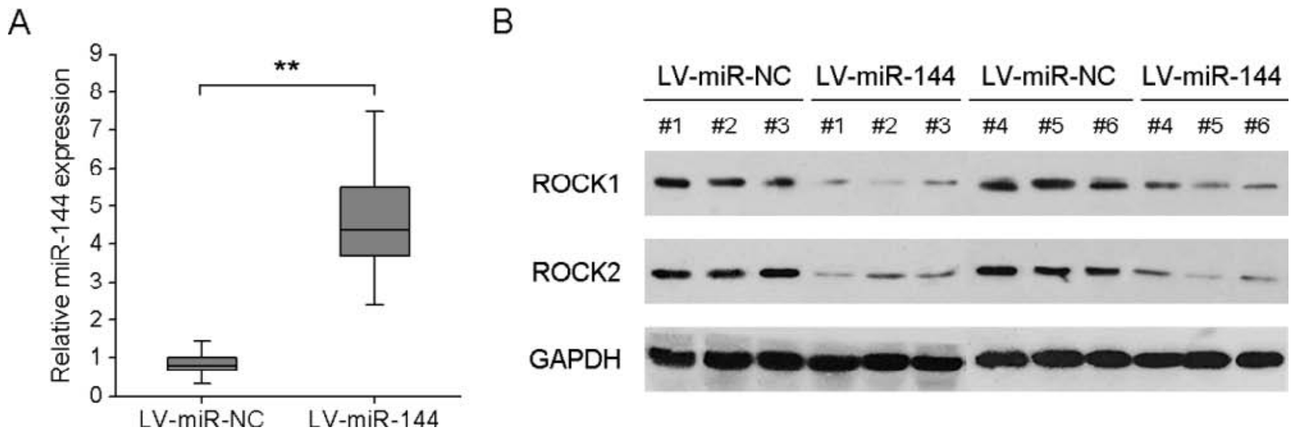

Supplementary Figure S2: miR-144 inhibited expression of ROCK1 and ROCK2 in xenograft tumor tissues. **A.** Expression of miR-144 in isolated tumors was analyzed by qRT-PCR. **B.** Western blot analysis of ROCK1 and ROCK2 protein levels in isolated tumors. $^{**}P < 0.01$.

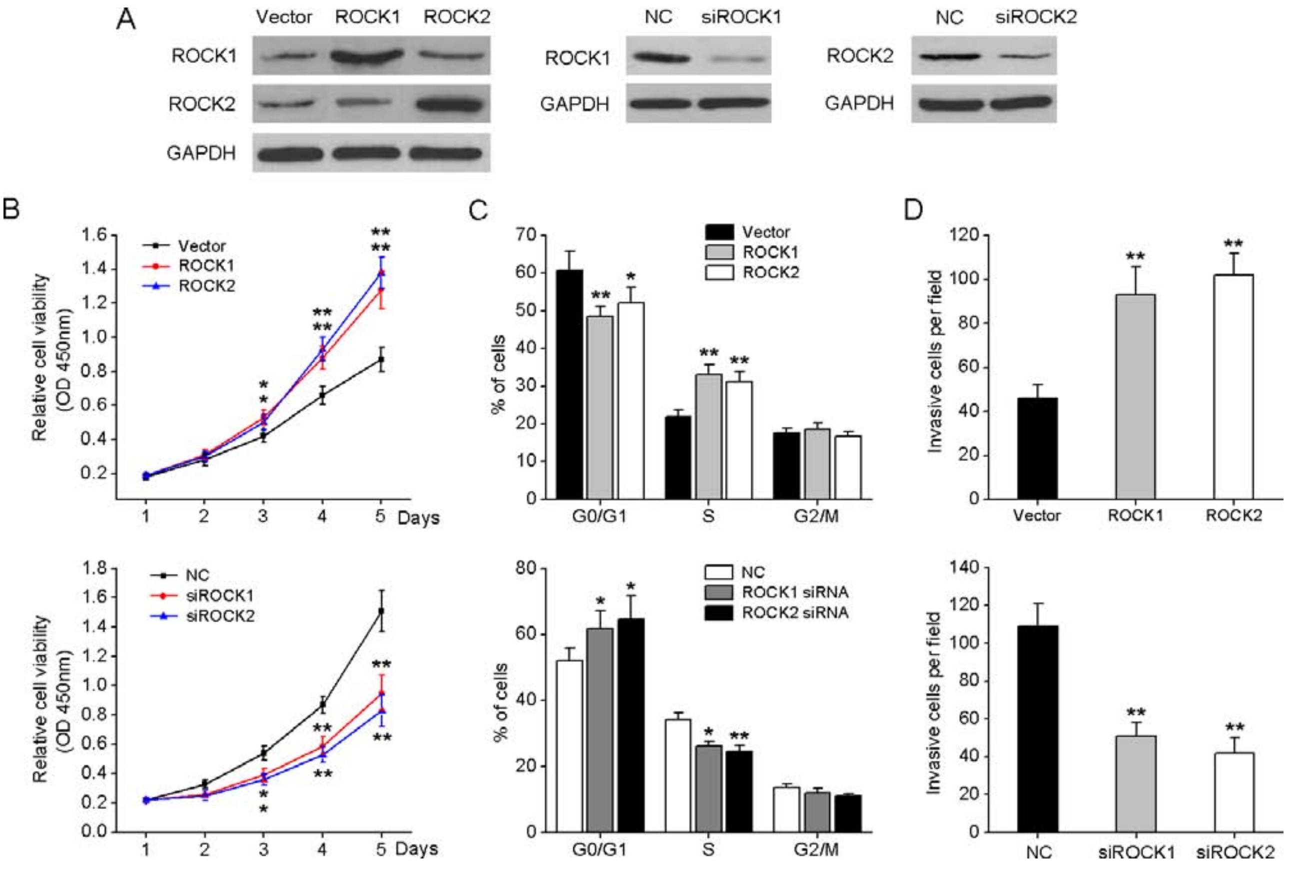

Supplementary Figure S3: Downregulation of *ROCK1* and *ROCK2* mediates miR-144's function in OS cells. 143B-miR-144 cells were transfected with empty pcDNA3.1 vector, ROCK1- or ROCK2-containing plasmid (left); 143B cells were transfected with siRNA against *ROCK1* or *ROCK2* (right). Western blot analysis of ROCK1 and ROCK2 expression is shown in **A.** Cell viability **B.** cell cycle distribution **C.** and cell invasion **D.** were measured with the CCK-8 assay, flow cytometry and transwell invasion assay, respectively. $^{*}P < 0.05$, $^{**}P < 0.01$.

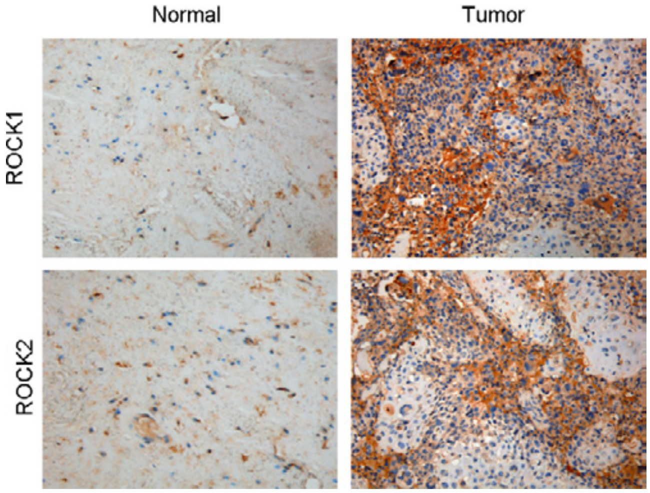

Supplementary Figure S4: Immunohistochemistry (IHC) analysis of ROCK1 and ROCK2 protein expression in OS. Representative images from IHC assays of 24 paired OS and adjacent normal bone tissues.

Supplementary Table S1. Summary of clinicopathologic variables

Clinical pathologicparameters	Number of Patients
**Gender**	
Male	39
Female	28
**Age (yrs)**	
< 18	31
≥ 18	36
**Location**	
Femur	43
Tibia	17
Other	7
**Tumor size (cm)**	
< 8	30
≥ 8	37
**Enneking stage**	
I	9
II	45
III	13
**Histologic grade**	
Low grade	9
High grade	58
**Metastasis**	
Present	13
Absent	54

Supplementary Table S2. Primers used for qRT-PCR

Primers		Nucleotide sequence
miR-144	RT-Oligo	5′-GTCGTATCCAGTGCAGGGTCCGAGGTATTCGCACTGGAT ACGACAGTACA-3′
	Sense	5′- TACAGTATAGATGA -3′
	Antisense	5′- GTGCAGGGTCCGAGGT -3′
U6 snRNA	RT-Oligo	5′-GTCGTATCCAGTGCAGGGTCCGAGGTATTCGCACTGGATACGACAAAATATG -3′
	Sense	5′- GCGCGTCGTGAAGCGTTC -3′
	Antisense	5′- GTGCAGGGTCCGAGGT -3′
ROCK1	Sense	5′- AAAAATGGACAACCTGCTGC -3′
	Antisense	5′- GGCAGGAAAATCCAAATCAT-3′
ROCK2	Sense	5′- CGCTGATCCGAGACCCT -3′
	Antisense	5′- TTGTTTTTCCTCAAAGCAGGA-3′
β-actin	Sense	5′- GCATCCTCACCCTGAAGTAC -3′
	Antisense	5′- GGCTGGGGTGTTGAAGGTCT -3′

Supplementary Table S3. Relationship between miR-144 expression and clinicopathologic features of OS patients

Clinical pathologic parameters	miR-144 expression		*P*-Value
	High	Low	
**Gender**			
Male	21	18	0.624
Female	13	15	
**Age (yrs)**			
<18	14	17	0.466
≥18	20	16	
**Location**			
Femur	23	20	0.648
Tibia	7	10	
Other	4	3	
**Tumor size (cm)**			
<8	19	11	0.087
≥8	15	22	
**Enneking stage**			
I	3	6	0.091
II	27	18	
III	4	9	
**Histologic grade**			
Low grade	3	6	0.305
High grade	31	27	
**Metastasis**			
Present	3	10	**0.033**
Absent	31	23	
